# Association of the Alumni and Officers Medical Department of the University of Buffalo

**Published:** 1875-01

**Authors:** 


### The Association of the Alumni and Officers of the Medical Department of the University of Buffalo.

*First Corporate Meeting. Public Exercises. Social Entertainment.*

The regular annual meeting of the Alumni Association of the Medical Department of the University of Buffalo, will be held Tuesday, February 23d, which is the regular Commencement Day for this year. The Incorporation Proceedings of the Association have been completed; Drs. Jas. P. White, Milton G. Potter, David E. Chace, C. C. Wyckoff, and Julius F. Miner, constituting the Board of Managers and Incorporators.

The meeting which opens at two o'clock in the afternoon of Commencement day at the Museum Hall in the College Building, will therefore be the first Corporate meeting of the Association, and its importance renders it worthy the particular effort of members in its attendance. The title designated in the Certificate of Incorporation, is that of "The Association of the Alumni and Officers of the Medical Department of the University of Buffalo," and by the Constitution and By-Laws which are to be presented for the action of the meeting, all Graduates of the Medical Department of the University of Buffalo, as also those who may constitute the Faculty or other Officers of the same, are considered Honorary Member of the Association, and on their compliance with the stated requirements can become regular active members. At this afternoon meeting, the President, T. D. Strong, M. D., of Westfield, N. Y., will deliver an Address. Voluntary communications will be in order also, while the business of the meeting will embrace matters of the perfecting of the organization, the adoption of a Constitution and By-Laws, miscellaneous business, and the election of officers for the coming year.

At seven and one-half o'clock in the evening, the regular exercises of Commencement, will occur in St. James Hall. After the ceremonies of Graduation, the charge to the Graduates will be given by Professor James P. White, and following this, an Alumni Address will be delivered by W. W. Potter, M. D., of Mount Morris, N. Y., of the Class of '59.

After the conclusion of the public exercises at the Hall, there will be an Association Entertainment and Supper at the Tifft House. Effort has been made by the College authorities, as well as by the Committee of the Alumni, in securing favorable time and opportunities for the business and pleasure of the Association, and it is anticipated that the session of the Society will be largely attended, and enjoyed by the members of the profession interested therein.

Though printed invitations are being sent by mail to the Alumni, still there are many whom the Committee may not reach in this way, and they therefore desire to give public and general invitation here, to all interested in the organization, to contribute by their presence and participation, to the pleasure and success of the session.

In order that the expenses of the collation may be defrayed by the participants, we have concluded to issue supper tickets at $1.50 each, which are to be obtained of the Secretary.

It is requested that all should report themselves to the Secretary of the Association, Wm. C. Phelps, M. D., at the afternoon session, or at the Secretary's office, No. 378 Main St., corner of Eagle, as soon as they arrive in town, in order that they may be registered and receive necessary instructions.

T. D. Strong,<br>

W. W. Miner,<br>

G. W. Pattison, } Committee.<br>

H. R. Hopkins,<br>

Wm. C. Phelps.

